# Enhancement of low-quality fetal electrocardiogram based on time-sequenced adaptive filtering

**DOI:** 10.1007/s11517-018-1862-8

**Published:** 2018-06-25

**Authors:** E. Fotiadou, J. O. E. H. van Laar, S. G. Oei, R. Vullings

**Affiliations:** 1 Department of Electrical Engineering, Eindhoven University of Technology, 5612 AP Eindhoven, Netherlands; 2 Department of Obstetrics and Gynaecology, Máxima Medical Center, 5504 DB Veldhoven, Netherlands

**Keywords:** Electrocardiography, Fetal ECG de-noising, Fetal ECG enhancement, Time-sequenced adaptive filter

## Abstract

Extraction of a clean fetal electrocardiogram (ECG) from non-invasive abdominal recordings is one of the biggest challenges in fetal monitoring. An ECG allows for the interpretation of the electrical heart activity beyond the heart rate and heart rate variability. However, the low signal quality of the fetal ECG hinders the morphological analysis of its waveform in clinical practice. The time-sequenced adaptive filter has been proposed for performing optimal time-varying filtering of non-stationary signals having a recurring statistical character. In our study, the time-sequenced adaptive filter is applied to enhance the quality of multichannel fetal ECG after the maternal ECG is removed. To improve the performance of the filter in cases of low signal-to-noise ratio (SNR), we enhance the ECG reference signals by averaging consecutive ECG complexes. The performance of the proposed augmented time-sequenced adaptive filter is evaluated in both synthetic and real data from PhysioNet. This evaluation shows that the suggested algorithm clearly outperforms other ECG enhancement methods, in terms of uncovering the ECG waveform, even in cases with very low SNR. With the presented method, quality of the fetal ECG morphology can be enhanced to the extent that the ECG might be fit for use in clinical diagnostics.

**Graphical abstract:**
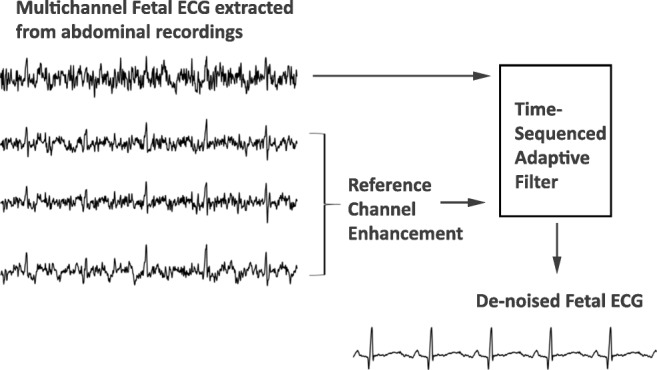
The extracted fetal ECG signals from non-invasive abdominal recordings still contain a substantial amount of noise. The time-sequenced adaptive filter provides a relatively accurate estimate of the underlying fetal ECG signal when the quality of the reference channels is enhanced prior to filtering.

The extracted fetal ECG signals from non-invasive abdominal recordings still contain a substantial amount of noise. The time-sequenced adaptive filter provides a relatively accurate estimate of the underlying fetal ECG signal when the quality of the reference channels is enhanced prior to filtering.

## Introduction

The fetal electrocardiogram (ECG) can be used to monitor the fetal condition from early pregnancy until delivery [24]. Analysis of its waveform provides information that can assist clinicians in making more appropriate and timely decisions during labor [17]. This can be applicable in the case of fetal hypoxia that occurs as a result of oxygen deprivation of the fetus during parturition. The condition is often accompanied by acidosis and is life-threatening unless prompt interventions are undertaken to restore well-oxygenated blood to fetus. Fetal hypoxia is found to be associated with changes in the ECG waveform [33]. The recording of the fetal ECG can be carried out by an invasive electrode or by placing skin electrodes on the maternal abdomen. Unfortunately, in abdominal recordings, the obtained signals are substantially contaminated by interferences and noise that vary depending on the gestational age, position of electrodes, skin impedance, etc. [24]. The main sources of interference include the maternal ECG, maternal and fetal muscle noise, powerline interference, baseline wander, movement artifacts, and multiple layers of dielectric biological tissues through which the electrical signals must pass. The signals from some of these interferences overlap in both time and frequency with the fetal ECG, complicating the extraction of the fetal ECG through conventional filtering techniques.

Despite the difficulties in acquiring fetal ECG signals non-invasively, a number of different techniques have been proposed in the literature such as neural networks [7, 21], wavelet-based methods [18, 23], singular value decomposition [22], blind source separation [6, 26, 42, 43], adaptive filtering [1, 14, 27, 39, 40], as well as combinations of different algorithms [15, 38, 41]. Clifford et al. [9] review the key achievements and the follow-up research generated as a result of the PhysioNet/Computing in Cardiology Challenge 2013 [35]. The challenge focused on fetal heart rate estimation and QT measurement in an automated way and managed to accelerate algorithm development in these areas [3, 5, 30, 36]. However, the extracted fetal ECG signal usually has a low signal-to-noise ratio (SNR) and additional processing is required to further enhance its quality. Beat-to-beat averaging [25] can be employed to improve the SNR of the signal. This approach, however, has the disadvantage that individual variations in pulse shape can be lost. In [39], an adaptive Kalman filter is developed that varies the number of complexes to be averaged according to the characteristics of the ECG signal. The filter is able to infer whether the ECG signal is corrupted by noise or dynamic variations and adapt the number of averages accordingly, in that way preserving the pulse variations. However, the filter is not extensively evaluated in fetal ECG signals with low SNR.

The proposed method focuses on the post-processing of the extracted fetal ECG signals, for enhancing their quality, based on adaptive filtering. Adaptive filters [11, 31, 40] have the ability to adjust their parameters autonomously and have been widely used to remove uncorrelated noise components when the noise characteristics are a priori unknown. Since the fetal ECG is a time-varying signal, adaptive filtering seems appropriate for estimating the fetal ECG. However, a least mean squares (LMS) adaptive filter [13] has been proven inadequate to fulfill this role due to the low SNR, complexity, and non-stationarity of the fetal ECG. Despite the substantial background noise removal achieved by the LMS adaptive filter, the signal distortion was too severe, causing relevant ECG morphology to disappear.

The fetal ECG signal is highly non-stationary, and a LMS filter is typically unable to track these rapidly varying non-stationarities. In [12], the time-sequenced adaptive filter (TSAF) has been suggested for the estimation of a class of non-stationary signals having a recurring statistical character and has shown good performance for detecting fetal ECG [1, 14]. The TSAF can be conceptualized as a bank of LMS adaptive signal enhancers that can achieve a rapidly varying impulse response. Fetal pulses differ among each other but have similar statistical properties. When the pulses are aligned according to a fiducial point, the statistics can be computed over the ensemble of pulses. The fiducial point at which the statistical properties of the signal renew is called the regeneration time. Each adaptive signal enhancer becomes an expert at filtering a specific signal segment between the regeneration times. The advantage of the method is that it does not require a priori knowledge of signal characteristics. However, an external input is needed to determine the regeneration times. In [1, 14], the R peak locations were detected to determine the regeneration times; Ferrara and Widrow [14] used a matched filter followed by peak detection, whereas Adam and Shavit [1] used a synchronized Doppler ultrasound signal.

The results of applying the TSAF to fetal ECG enhancement, presented in [1, 14], show that, in case of poor SNR, the characteristic waves of the ECG cannot be distinguished after filtering. Moreover, as mentioned in [1], when the input SNR is relatively low, the effect of the filter’s regeneration time can be seen in some cardiac cycles. This paper presents an improvement of the TSAF for fetal ECG enhancement and demonstrates the feasibility of time-sequenced adaptive filtering for enhancing the quality of the fetal ECG to the extent that the morphology of the signal is preserved. This improvement involves the enhancement of the reference inputs prior to their use in the TSAF, yielding an increase in the SNR of the TSAF output, and the elimination of aforementioned effects of the regeneration time via the use of overlapping filters.

## Materials and methods

### The time-sequenced adaptive filter

**Fig. 1:**
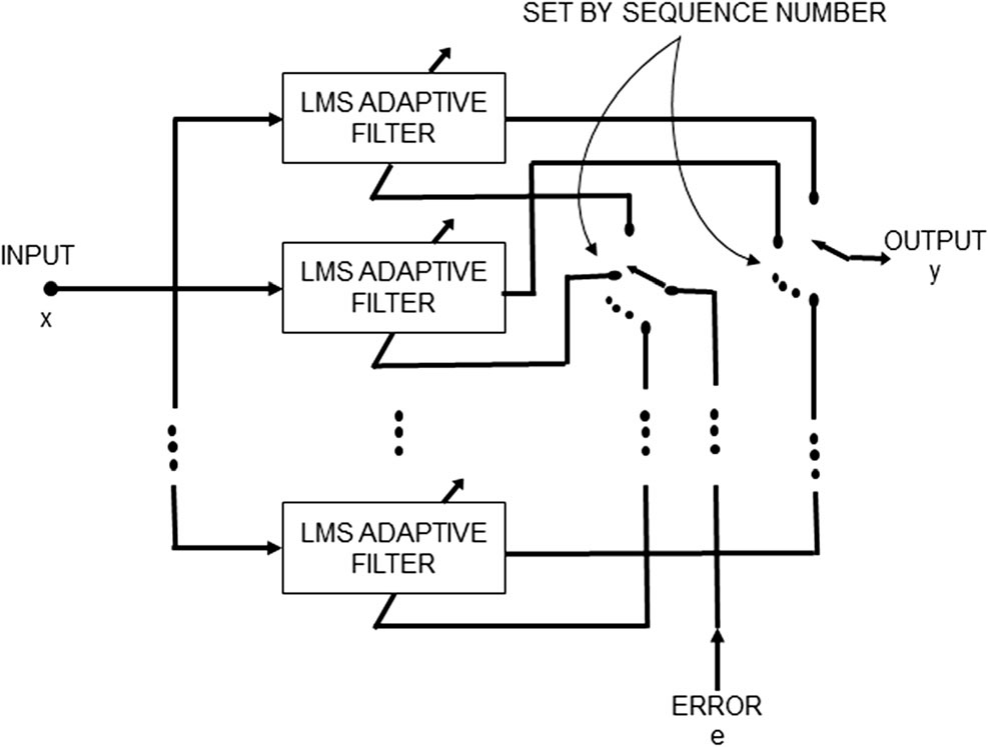
Structure of the time-sequenced adaptive filter. The filter is realized as a bank of LMS adaptive filters. The sequence number determines which LMS filter is used at each point in time

The TSAF provides a separate LMS enhancer for each signal sample in an ECG cycle, as opposed to the original adaptive signal enhancer [11] that consists of a single LMS filter. Thus, there are a number of adaptive filters equal to the length of an ECG complex and each one is updated in every ECG cycle. The structure of the filter is depicted in Fig. 1. An external input, known as the sequence number, determines which LMS enhancer to be used at each point in time. The LMS adaptive filter is a stochastic gradient-based algorithm that utilizes the gradient vector of the filter tap weights to converge to the optimal Wiener solution [40]. Each iteration of the LMS algorithm consists of the following steps:

1. Calculation of the filter output

1 $$ y(n)={\mathbf{w}}^T(n)\mathbf{x}(n) $$

where **x**(*n*) is the filter input and **w**(*n*) is the filter weights. The size of the vectors is equal to the filter order (*P*).

**Fig. 2:**
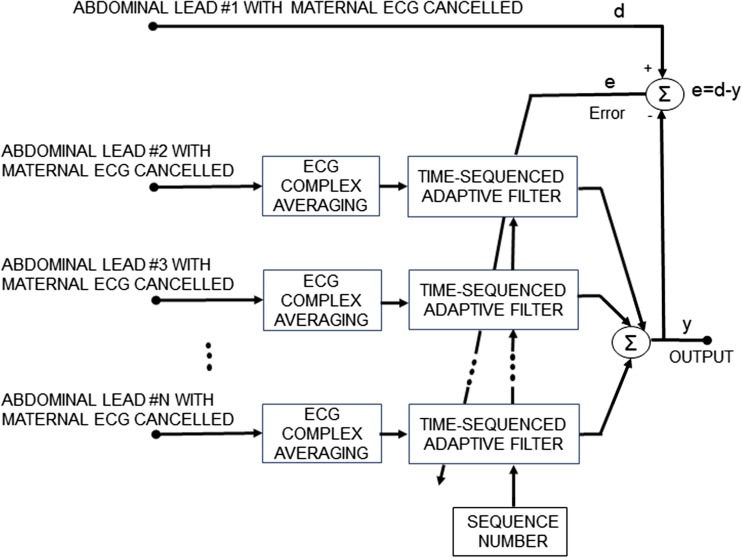
Block diagram of the proposed *N*-channel time-sequenced fetal ECG enhancement method. The reference channels (no. 2–no. *N*) are enhanced prior to filtering. The filter output is an estimate of the de-noised fetal ECG signal of channel 1

2. Estimation of the error signal which is needed to update the filter coefficients in the next step

2 $$ e(n)=d(n)-y(n) $$

where *d*(*n*) corresponds to the *n*th sample of the desired solution (in our case, the signal to be enhanced; see Fig. 2).

3. Update of the filter weights for the next iteration

3 $$ \mathbf{w}\left(n+1\right)=\mathbf{w}(n)+\mu e(n)\mathbf{x}(n) $$

where *μ* is a step-size parameter.

The weights (**w**(*n*)) are initialized as zero. The step-size parameter controls the rate at which the weights change. The choice of this parameter is critical for balancing the convergence of the filter versus its stability. Selecting a step size that guarantees stability and ensures fast enough convergence is difficult due to the sensitivity of the LMS algorithm to scaling of its input [11]. To resolve this issue, in this work, we use the normalized least mean squares (NLMS) filter instead of the LMS. NLMS is an extension of the standard LMS algorithm with the difference that it uses a time-varying step size, yielding a faster convergence as opposed to the LMS algorithm [10]. The step size used by the NLMS filter is defined as:

4 $$ {\mu}^{\prime }(n)=\frac{\overline{\mu}}{\mathbf{x}{(n)}^T\mathbf{x}(n)} $$

where $\overline{\mu}$ is a scalar which allows for a change in the adaptation speed. The normalization of the step size with the power of the input signal (**x**(*n*)) makes the algorithm insensitive to scaling.

### Proposed method

#### Enhancement of reference signals

In this study, we have performed pre-processing and maternal ECG removal following the method developed by Varanini et al. [36]. The residual signals comprise of fetal ECG and a significant amount of remaining noise. Typically, the fetal ECG components among different channels are correlated, whereas some remaining noise components such as muscle noise are mostly uncorrelated. We propose the use of an augmented TSAF (aTSAF) to enhance the quality of multichannel fetal ECG by attenuating the uncorrelated noise (Fig. 2). The channel to be de-noised is considered as the primary channel and the other channel(s) as the reference channel(s). In Fig. 2, the use of channel 1 as primary channel is just an example. Every channel that we wish to de-noise can be considered as primary channel. The reference channels should be correlated with the primary one and should have sufficiently high SNR to yield satisfactory performance of the TSAF. To ensure an acceptable SNR of the reference inputs, in this work, we enhance the quality of the reference channels by ensemble averaging, prior to using them in the TSAF. The ensemble averaging includes the detection of the R peak locations by the method described in [36], the alignment of successive complexes according to these locations, and the ensemble averaging of aligned ECG complexes. In the ensemble averaging, we used the ECG of 30 consecutive heartbeats to preserve clinically relevant variations in the ECG and, at the same time, produce substantial enhancement of the ECG [33].

#### Scheme for faster algorithm convergence

After the reference channels have been enhanced, the TSAF combines them via a dynamic linear combination, where the weights in this linear combination are optimized for minimizing the error between the TSAF output and the primary input. To maintain a high rate of convergence, without risking instability of the process, a scheme is employed as described in the work of Cano et al. [8]. According to it, the filter weights (**w**(*n*)) are updated not just in the prior ECG cycle but also in the current cycle where they are used for filtering. The scheme makes the assumption that immediate neighboring data samples are highly correlated and can be used to approximate each other; thus, the weights can be updated in the cycle being filtered. It is only employed for a given number of ECG cycles to assist the algorithm to achieve faster convergence.

#### Reduction of the regeneration time effect

The TSAF requires the knowledge of the location of the fetal ECG fiducial points in order to determine the regeneration times. The sequence number at each regeneration time is (re)set to 1 and increases by 1 for each data sample. Since the fetal PR interval is approximately 100 ms [37], the regeneration times are chosen to occur 160 ms before the detected R peaks. In such a way, the start of the sequence occurs before the P wave starts. The sequence length defines how many adaptive signal enhancers constitute the TSAF. Ideally, we want the sequence length to be equal to the ECG complex length but this varies from cycle to cycle. Hence, we defined the sequence length to be 110% of the mean interval between the fetal R peaks. In this way, it is ensured that in most cases, the sequence length is bigger than the length of the ECG cycle, allowing all the samples in the cycle to be processed by a separate adaptive filter. This implies, however, that in most cases, there are overlapping signal parts that are filtered twice. These parts are smoothed by averaging the contributions of both overlapping complexes. To be more specific, the contribution of the first ECG complex is gradually reduced while the contribution of the second complex is gradually increased. Inevitably, there are cases where the length of the sequence is smaller than the size of the ECG complex. In this situation, gaps exist between successive complexes. The samples in these gaps are filtered by the same LMS adaptive filter that was used for the last sample in the sequence. The weights of this filter are updated only once in the current cycle. This guarantees that all the filters in a cycle converge with similar speed. By selecting the start and length of the sequence in the way described before, we avoid that the characteristic waves of the ECG complex fall outside the borders of the sequence in cases of ECG complexes longer than the sequence length. This is very important since the waves contain all the useful information about the physiological state of the fetus. Following the described approach, the regeneration time effect noticed in [1] can be significantly reduced.

### Data description

The fetal ECG signals, even after the maternal ECG has been removed, are still affected by noise. It is hence impossible to have a gold reference (i.e., clean signal) that can be used to quantitatively validate the proposed algorithm. As a surrogate, in this study, the proposed method is extensively validated based on simulated signals of the Fetal ECG Synthetic Database (FECGSYNDB) of PhysioNet [2, 16]. To illustrate the potential of our method on real data, we have included results obtained from data in the Abdominal and Direct Fetal Electrocardiogram Database of PhysioNet [20].

The FECGSYNDB consists of 1750 synthetic abdominal signals with 34 channels, sampling frequency of 250 Hz, and duration of 5 min. The database includes ten simulated pregnancies with seven different physiological events as shown in Table 1. The signal-to-noise ratio of the simulated signals varies from 0 to 12 dB in steps of 3 dB. In each simulation, signals are generated five times for statistical purposes. In this work, we use six channels (i.e., 1, 8, 11, 22, 25, and 32) for evaluation of our algorithm, as suggested by Andreotti et al. [2]. The signals simulating twin pregnancy (fifth case, Table 1) are excluded from our analysis, since the proposed algorithm is not developed to handle this case.

**Table 1:** Description of the seven cases of physiological events of the synthetic signals of the Fetal ECG Synthetic Database

Case	Description
Baseline	Abdominal mixture (no noise or events)
Case 0	Baseline (no events) + noise
Case 1	Fetal movement + noise
Case 2	Acceleration or deceleration of maternal and fetal heart rate + noise
Case 3	Uterine contraction + noise
Case 4	Ectopic beats for both fetus and mother + noise
Case 5	Twin pregnancy + noise

The Abdominal and Direct Fetal Electrocardiogram Database contains multichannel fetal electrocardiogram (FECG) recordings obtained from five different women in labor, between 38 and 41 weeks of gestation. Each recording contains four signals acquired from the maternal abdomen and one scalp ECG signal. The recordings have a duration of 5 min and are sampled at 1000 Hz.

**Fig. 3:**
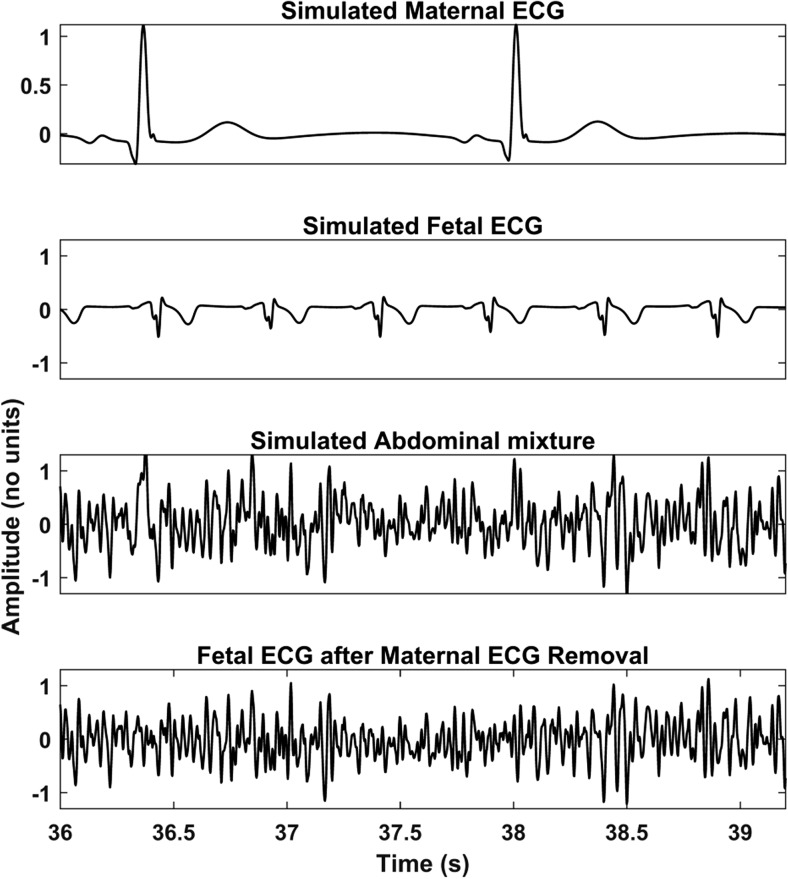
An example of simulated signals of the FECGSYNDB. The first row shows the simulated maternal ECG; the second the simulated fetal ECG; the third the abdominal mixture, where noise is also added; and the last the extracted fetal ECG after the method of Varanini et al. [36] is applied. The displayed segment corresponds to the third channel (third of the six channels in use) of the tenth simulated pregnancy, SNR 0, case 0, and second repetition

**Fig. 4:**
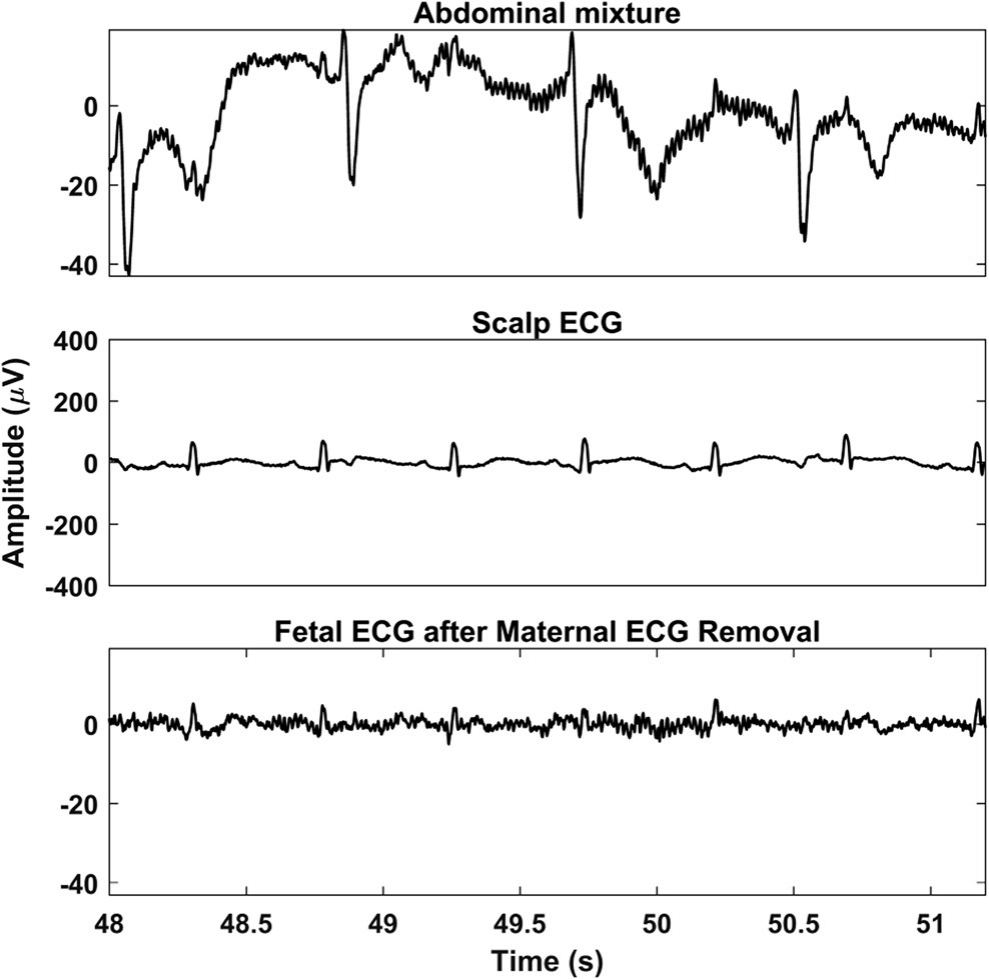
An example of real signals of the Abdominal and Direct Fetal Electrocardiogram Database. The first row shows a segment of the abdominal mixture of channel 1 for recording “r07.” The second displays the simultaneously recorded scalp fetal ECG, while the last the extracted fetal ECG after the method of Varanini et al. [36] is applied

The signals of both databases are pre-processed before the proposed method is applied to them. First, the signals are resampled to 500 Hz to have a common reference. Then, the open-source algorithm of Varanini et al. [36] is applied to the signals. According to this algorithm, first, the baseline wander and the powerline interference are removed. After that, the maternal ECG is estimated through independent component analysis (ICA) and singular value decomposition and subsequently subtracted from the signals. Finally, a second ICA is employed to enhance the fetal ECG signal and two QRS detectors are applied in forward and backward directions to obtain the R peak locations. Figures 3 and 4 show an example of simulated data and real data respectively together with the result of the aforementioned pre-processing. In both cases, the extracted fetal ECG after the maternal ECG removal contains a significant amount of remaining noise.

### Performance measure

The performance of the method is assessed based on the SNR improvement in the filter output when compared to the input signal. The metric is computed as follows:

5 $$ {\mathrm{SNR}}_{\mathrm{imp}}\left[ dB\right]=10{\log}_{10}\frac{\sum \limits_{n=1}^M{\left|d\left[n\right]-z\left[n\right]\right|}^2}{\sum \limits_{n=1}^M{\left|y\left[n\right]-z\left[n\right]\right|}^2} $$

where *z*[*n*] denotes the original clean fetal ECG signal, *d*[*n*] the noisy signal, *y*[*n*] the enhanced one, and *M* the length of the signals. The metric is measured for each channel and subsequently summed over all ECG channels and over all signals.

The proposed method is compared to several other ECG enhancement algorithms. The first one is a wavelet de-noising method that decomposes the signal, thresholds the detail coefficients, and reconstructs the signal to obtain its enhanced version. The 6-order Daubechies wavelet is selected as a mother wavelet because of its similarity to an actual ECG. A fixed threshold is used which is estimated by the minimax principle [34]. Secondly, our algorithm is compared to the ensemble averaging over 30 consecutive ECG complexes. The next reference method is the adaptive Kalman filter described in [39], where the number of the ECG complexes to be averaged is adapted according to the signal characteristics. An additional comparison is made with the multichannel NLMS adaptive signal enhancer [11] and with the NLMS adaptive signal enhancer for which the reference channels are enhanced via our suggested averaging method (we will refer to this method as augmented NLMS, aNLMS), as described in Section 2.2.1. Finally, our method is also compared to the TSAF without pre-processing of the reference channels but including compensation for regeneration time effects.

## Results

### Parameter optimization

The proposed algorithm has few parameters that must be chosen: the parameter $\overline{\mu}$ that is used in the calculation of the step size of NLMS algorithm and *P*, the length of the adaptive filters. The value of $\overline{\mu}$ is critical for the algorithm’s performance since a wrong choice will restrain the algorithm from convergence. However, once chosen appropriately, this parameter does not need further readjustments because of the scaling tolerance of the NLMS algorithm. The choice for *P* is less critical.

In order to select these two parameters, the simulated signals of the FECGSYNDB are separated in a training and a test set. The first five simulated pregnancies are used as training data while the last five as test. The training set is used to optimize the parameter values, while the test set is used to evaluate the performance of the algorithm. An iterative procedure is performed over several parameter values, and the ones that produce the highest SNR improvement in the training set are finally selected. For $\overline{\mu}$, a search is performed between the values 0.0005 and 0.1 with eight logarithmic steps. Regarding the filter length, the values of 50 and 100 to 800 with steps of 100 are used. For TSAF, NLMS, and aNLMS, the parameter values are selected similarly. The parameter values that produce the best results for all the algorithms are 0.005 for $\overline{\mu}$ and 100 for *P*.

### Evaluation on the Fetal ECG Synthetic Database

Our method is evaluated on data from the FECGSYNDB described in Section 2.3. Since the proposed algorithm requires the knowledge of the fetal R peaks, only the signals for which at least 80% of the fetal R peaks are detected within an error of 50 ms from the actual R peak [4] are included in the evaluation of the algorithm. Table 2 shows the number of signals with successful R peak detection for each of the different cases of the FECGSYNDB. The numbers correspond to the simulated pregnancies 6–10. As expected in the baseline case, where no noise is added to the abdominal mixture, the extracted fetal ECG signals are of relatively good quality and the R peak detection succeeds in almost all the cases. Case 4, where ectopic beats are simulated, is the most challenging one and the R peak detection succeeds only in 30 out of 125 occurrences.

**Table 2:** The number of the signals with successful R peak detection for each of the different cases of the FECGSYNDB (125 synthetic signals per case of the test-simulated pregnancies 6–10)

Case	Number of signals
Baseline	121
Case 0	94
Case 1	106
Case 2	84
Case 3	78
Case 4	30
Total	513

**Fig. 5:**
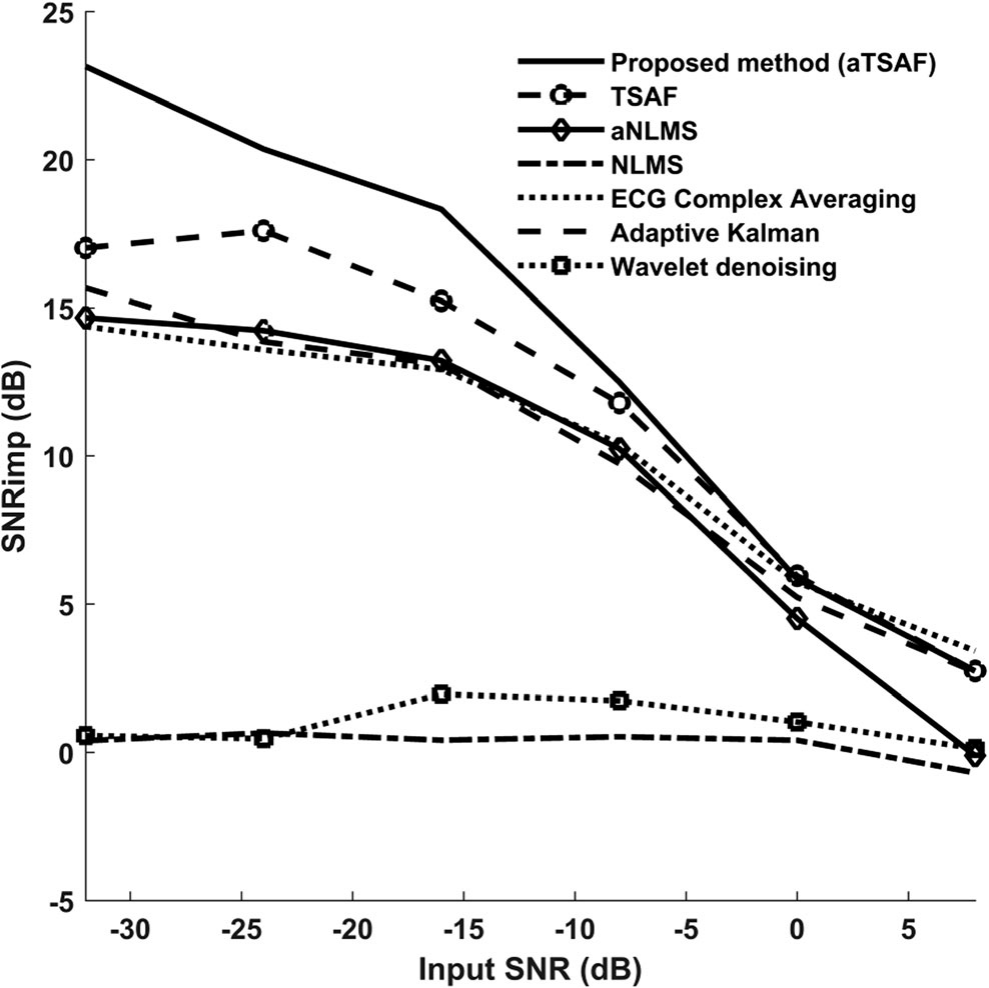
Performance of different fetal ECG enhancement algorithms in comparison with the proposed algorithm by means of improvement in the SNR of the output when compared to the SNR of the input signal. The results correspond to the average over all channels and cases for the synthetic signals of the FECGSYNDB (simulated pregnancies 6–10)

Figure 5 illustrates the performance of the proposed method in comparison to the de-noising algorithms mentioned in Section 2.4 for the different SNR values of the input signals. The input SNR refers to the SNR of the signals after the maternal ECG has been removed, and this SNR is averaged over all channels and measurements. From Fig. 5, it can be observed that, for very low SNR values of the input signals, the proposed algorithm clearly outperforms the other algorithms. For input SNR less than − 15 dB, the suggested method provides an additional SNR improvement of at least 3 dB over the other best-performing methods. For high SNR values (more than 0 dB), the conventional TSAF algorithm produces a similar result with our algorithm since there is no need to filter the reference channels. It is worth observing the increase in performance of the aNLMS algorithm over the conventional NLMS. Without our proposed pre-processing of reference signals, NLMS performs significantly worse. From Fig. 5, it can be seen that the averaging of ECG complexes produces good results when the SNR is higher than − 10. However, for lower SNR values of the input signals, the SNR improvement is significantly lower than that of the proposed algorithm. Apparently, different ECG segments should be considered separately to truly enhance the ECG. Plain averaging works on the entire ECG complex and treats it as a whole. On the other hand, our method uses different filtering schemes for different parts of the ECG.

**Fig. 6:**
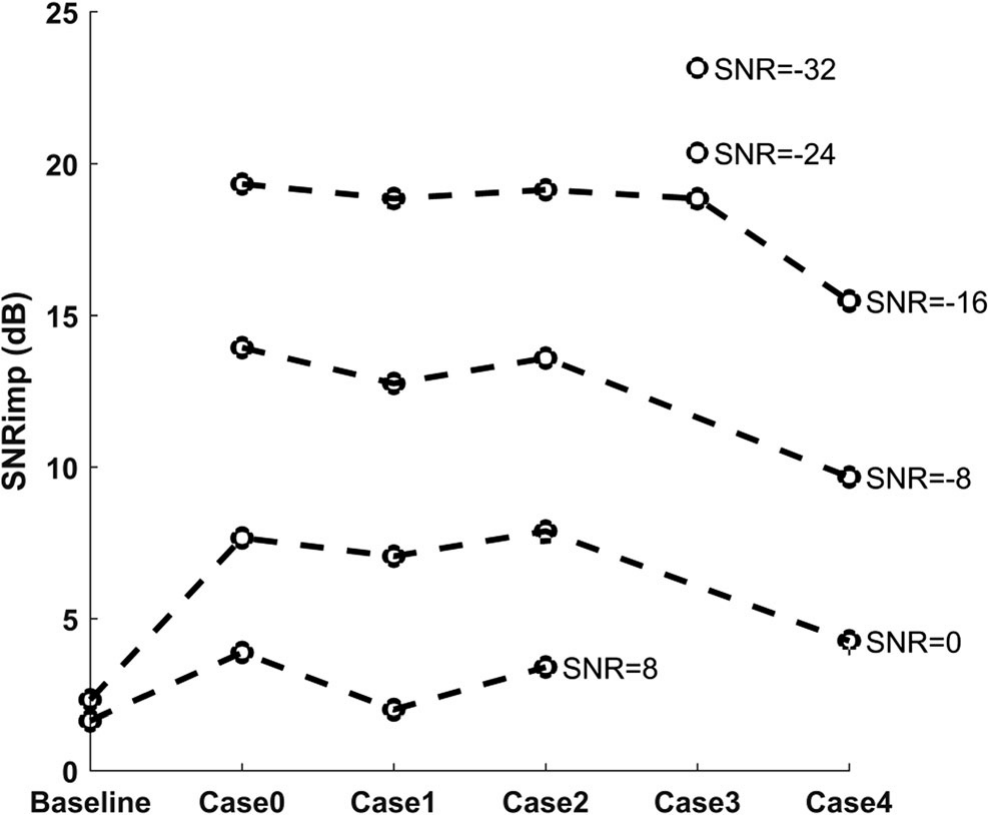
Performance of the proposed aTSAF algorithm for each of the different cases and the input SNR of the synthetic signals of the FECGSYNDB. The input SNR corresponds to the average of all channels and after the maternal ECG is removed. The “o” circles show the performance only for the cases where there are available input signals with the specific SNR (− 32 to 8 dB)

Figure 6 depicts the performance of the suggested method for each of the different cases separately and for all the ranges of available input SNRs. The input SNR values are clustered in six groups of values from − 32 to 8 dB in steps of eight. Input signals with SNR from all these groups are not available for every case, since the SNR is measured after the maternal ECG is removed from the simulated signals. As we can see in Fig. 6, the efficiency of aTSAF for each specific input SNR is similar for the different cases apart from case 4 and the baseline case. Case 4, as mentioned before, is a challenging case that simulates the presence of ectopic beats. In this case, abrupt changes in the morphology of the ECG signals occur, for a short amount of time, making it relatively impossible for the algorithm to adapt fast enough. In this case, our algorithm adapts to regular heartbeats but is incapable of tracking the sudden changes. Regarding the baseline case, less improvement in SNR is achieved compared to the other cases. The reason is that this case does not contain added noise that can be removed by the aTSAF.

**Fig. 7:**
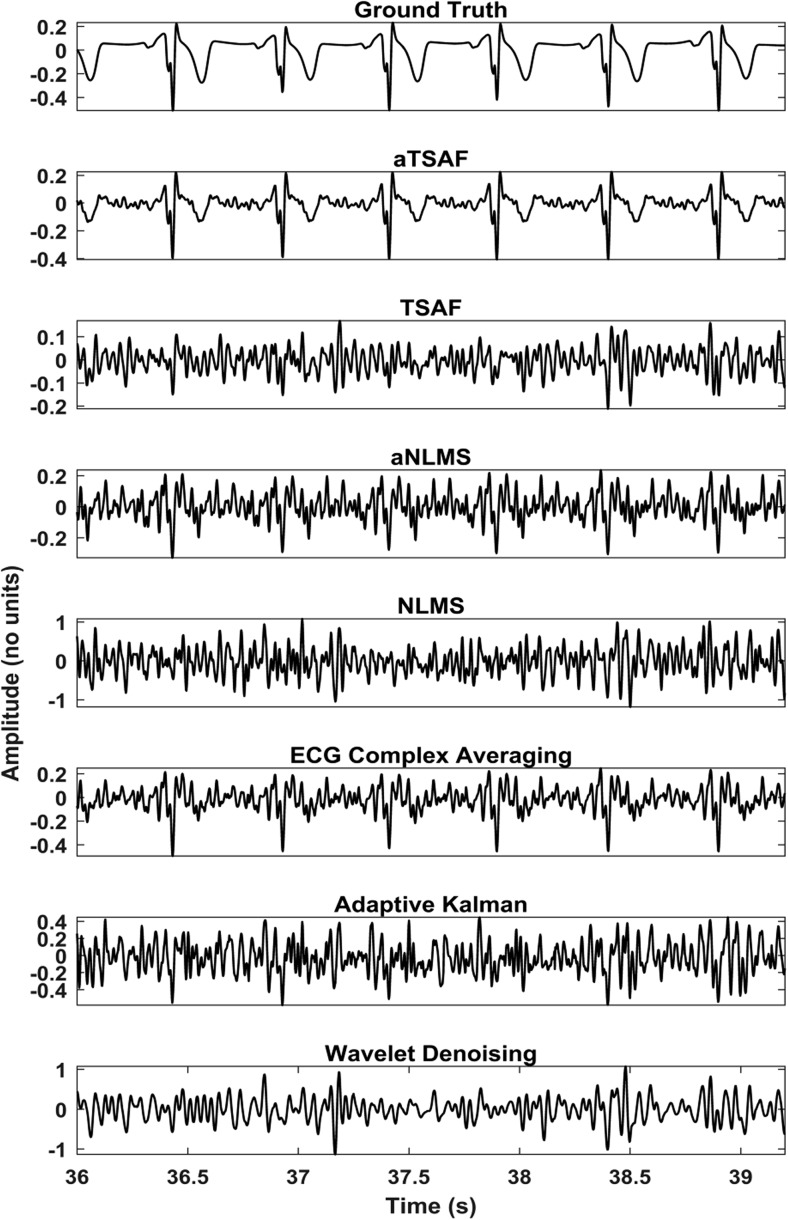
Enhancement result of the different algorithms for the simulated signals described in Fig. 4. In the first row, the simulated clean fetal ECG signal is displayed for comparison. Only the proposed method (aTSAF) provides a high-quality result in this challenging case of low-SNR input signals (− 18 dB on average)

The results of processing the simulated signals, which are described in Fig. 3, by our aTSAF method are illustrated in Fig. 7. For the depicted recording, the average SNR of the six channels after the maternal ECG has been removed is − 18 dB. The figure demonstrates that the proposed aTSAF method is capable of suppressing the noise to the extent that the individual waves in the ECG complex become distinguishable. Moreover, there is virtually no distortion of the ECG signal. In this particular case, the suggested algorithm clearly outperforms the other methods. The additional step of enhancing the quality of reference channels—which is the main contribution of this study—makes a significant difference in the performance of the TSAF algorithm, especially for cases with low-quality signals. For the other algorithms, the ECG complex averaging and the aNLMS produce the best results but show still a significant amount of noise in the filtered signals. For the other methods, the morphological information of the ECG cannot be seen.

### Evaluation on the Abdominal and Direct Fetal ECG Database

**Fig. 8:**
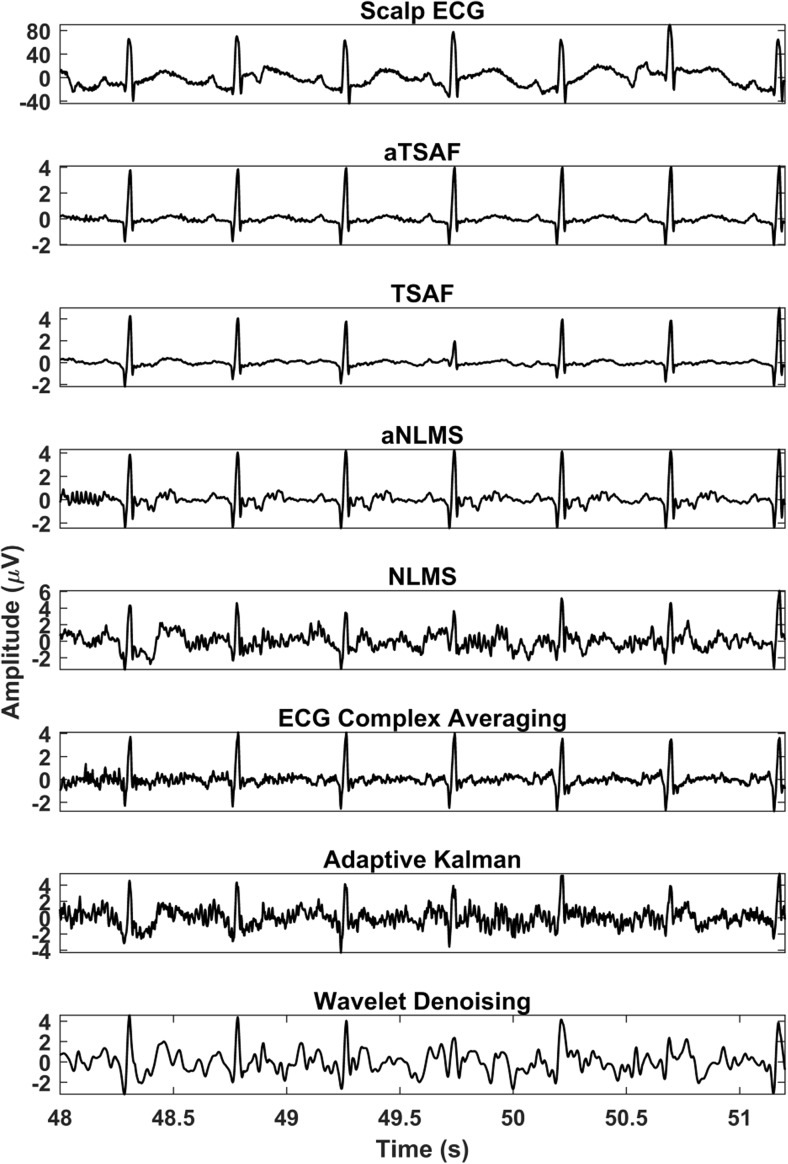
Comparison of the enhancement results of the different algorithms for channel 1 of recording “r07” of the Abdominal and Direct Fetal Electrocardiogram Database. In the first row, the simultaneously recorded scalp ECG is presented. The noisy signal is shown in Fig. 5. In the output of the proposed method (aTSAF), the characteristic ECG waves are clearly visible and correspond well to those of the scalp ECG

In this subsection, the performance of the same algorithms evaluated in Section 3.2 is evaluated on actual data from the Abdominal and Direct Fetal ECG Database. Concerning the algorithm parameters, the same parameters that were optimized in the FECGSYNDB are used. In the real data, because of the lack of ground-truth data, the performance of the various methods is evaluated qualitatively, as opposed to quantitatively for the simulated data. To demonstrate the potential of abdominal ECG recordings, we have shown the scalp ECG, after high-pass filtering for baseline wander removal, as well in our figures (see, e.g., Fig 8). It should be noted that the scalp ECG is a different ECG lead than the abdominal leads and that they should not be identical, even in case of perfect enhancement. Nevertheless, the individual ECG segments should coincide between abdominal leads and scalp lead.

Figure 8 illustrates the results of various algorithms for the fetal ECG enhancement of channel 1 of recording “r07,” which was already depicted in Fig. 4. As seen in Fig. 8, the ECG signal filtered by the proposed aTSAF algorithm is relatively free from noise and the individual waves correspond well to those in the scalp ECG. Pre-processing of the reference channels appears to have a substantial contribution to the performance of both the TSAF and NLMS algorithms, making even the small waves distinguishable. Without our proposed pre-processing, these characteristic waves are often not visible, either because they were suppressed by the filter or because the noise was not suppressed enough.

**Fig. 9:**
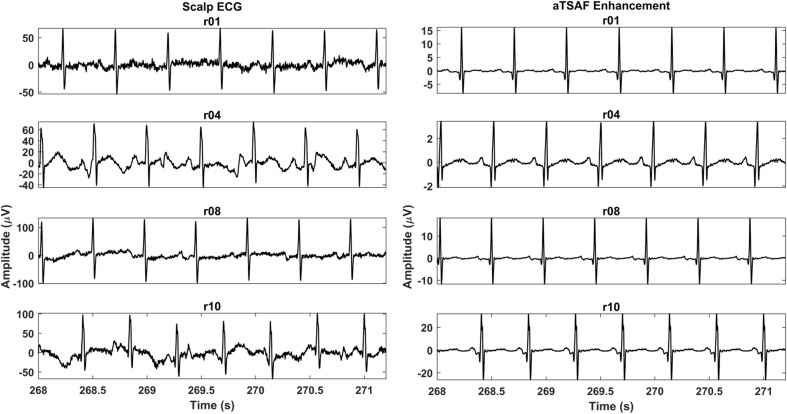
The enhancement result of the proposed method (aTSAF) for the recordings “r01,” “r04,” “r08,” and “r10” of the Abdominal and Direct Fetal Electrocardiogram Database. In the left column, the corresponding scalp ECGs are displayed. In all cases, the enhanced signals are clean and the morphological signal characteristics are similar to those of the scalp ECGs

Figure 9 demonstrates the results of the processing of the four other recordings of the Abdominal and Direct Fetal ECG Database with the proposed algorithm. The corresponding scalp ECGs are presented together with the results for channel 1 of each recording. In all cases, the suggested method produces a relatively clean ECG signal with morphology that corresponds relatively well to that of the scalp ECG, especially for the P wave and QRS complex.

## Discussion

A method is presented here for post-processing of the extracted fetal ECG after the maternal ECG is removed. In most cases, the extracted fetal ECG still contains a substantial amount of noise that impedes the interpretation of the morphology of the ECG signal by clinicians. Usually, the QRS complex can be detected without further processing of the fetal ECG, due to the high amplitude of the R peak. However, the smaller waves, like the P and T waves, can often not be readily distinguished. Thus, post-processing of the extracted fetal ECG signal, to enhance its quality, becomes of paramount importance. For this purpose, in the proposed method, the TSAF is improved by increasing the quality of the reference channels and by exploiting overlapping filters to minimize the effect of regeneration times. The improved filter is found to be effective in reducing major components of noise. But more than that, the main contribution of the filter is that after the filtering, the signal morphology is retained to the extent that even the small signal waves can be visually distinguished. As a plus, the method is relatively insensitive to the choice of parameters and, as such, is rather generally applicable.

A limitation of the method is that an estimate of the R peak locations is required to determine the regeneration times of the filter. However, this does not necessarily impede the use of our filter. First of all, a lot of valuable work has been already done in the area of fetal R peak estimation [3, 5, 30, 32, 36] with very promising results. Second, in a practical application, the user could be informed to, in case of missing R peaks, distrust the output of the filter. Also, there is the possibility of using other measurement modalities to yield the regeneration times, like synchronous Doppler echocardiography as was proposed by Adam and Shavit [1].

Another shortcoming of the method is in cases of arrhythmia and ectopic beats. In these cases, sudden and brief changes are happening to ECG signal morphology and the filter is not able to adapt fast enough. As a consequence, it will only adapt to the morphology of regular beats and would be incapable of tracking the abrupt changes. This effect is also caused because of the averaging of ECG complexes for the enhancement of reference channels. Because of the averaging, brief variations in the ECG morphology are filtered out. However, without this enhancement step, the TSAF is unable to efficiently remove the noise. Moreover, the target application of this work is to enhance the fetal ECG quality for detecting hypoxia. The STAN method [33] that is used for fetal monitoring with an invasive electrode averages 30 consecutive heartbeats for ECG signal enhancement. For a typical fetal heart rate of about 140 beats per min (BPM), 30 heartbeats correspond to a time interval of 13 s. Transient or structural changes in the ECG waveform that have clinical relevance with respect to developing hypoxia are hypothesized in the STAN methodology to occur over longer time scales. Consequently, averaging 30 consecutive ECG complexes preserves clinically relevant variations in the ECG and, at the same time, yields a substantial enhancement of signal quality. Based on the same reasoning, we also average 30 consecutive ECG complexes to enhance our reference channels, while maintaining relevant information for detecting hypoxia.

The high-quality signals that the proposed method delivers can give the opportunity to clinicians to measure the exact timing of different morphological features of the ECG signal. Besides, it can facilitate and advance the research towards automated detection of fetal ECG intervals and segments. Extracting morphological features from the ECG signal allows for the estimation of the well-being of the fetus. Fetal acidosis is known to affect ECG morphology [17, 19], while asphyxia of the fetus is thought to be associated with changes in the P wave, PQ interval, and ST segment [29]. Moreover, fetal growth might also influence the timing of ECG waves [28].

## Conclusion

This paper presented a method to improve the performance of the time-sequenced adaptive filter for fetal ECG enhancement. In the proposed method, the quality of the reference channels is enhanced prior to filtering via ensemble averaging of multiple consecutive ECG complexes. The evaluation of our filter, on both simulated and real fetal ECG signals, shows that the proposed algorithm outperforms the conventional time-sequenced and the NLMS adaptive filtering techniques. Our results indicate that pre-processing of the reference channels provides a more accurate estimate of the underlying fetal ECG signal. The proposed algorithm can reveal the characteristic waves of the fetal ECG signal, even in cases with relatively low SNR. A limitation of our method is that, in case of rapidly changing ECG morphology, for instance in the presence of ectopic beats, our algorithm is unable to track these changes, yielding a suboptimal performance. Moreover, an estimate of the position of the fetal pulse locations is required to determine the regeneration times of the filter. Future work could focus on performing automated extraction of relevant morphological features such as PR intervals, QT intervals, and ST segments.
